# Do atmospheric events explain the arrival of an invasive ladybird (*Harmonia axyridis*) in the UK?

**DOI:** 10.1371/journal.pone.0219335

**Published:** 2020-01-15

**Authors:** Pilvi Siljamo, Kate Ashbrook, Richard F. Comont, Carsten Ambelas Skjøth

**Affiliations:** 1 Meteorological Research, Finnish Meteorological Institute, Helsinki, Finland; 2 School of Science & the Environment, University of Worcester, Worcester, England, United Kingdom; University of Delhi Department of Environmental Studies, INDIA

## Abstract

Species introduced outside their natural range threaten global biodiversity and despite greater awareness of invasive species risks at ports and airports, control measures in place only concern anthropogenic routes of dispersal. Here, we use the Harlequin ladybird, *Harmonia axyridis*, an invasive species which first established in the UK from continental Europe in 2004, to test whether records from 2004 and 2005 were associated with atmospheric events. We used the atmospheric- chemistry transport model SILAM to model the movement of this species from known distributions in continental Europe and tested whether the predicted atmospheric events were associated with the frequency of ladybird records in the UK. We show that the distribution of this species in the early years of its arrival does not provide substantial evidence for a purely anthropogenic introduction and show instead that atmospheric events can better explain this arrival event. Our results suggest that air flows which may assist dispersal over the English Channel are relatively frequent; ranging from once a week from Belgium and the Netherlands to 1–2 times a week from France over our study period. Given the frequency of these events, we demonstrate that atmospheric-assisted dispersal is a viable route for flying species to cross natural barriers.

## Introduction

Invasive alien species are widely recognised as agents of global biotic homogenisation and thus as one of the main challenges to future global biodiversity [1]. Species introduced outside their natural range and which have detrimental effects on native species are known as Invasive Alien Species (IAS) and are recognised as a significant component of environmental change worldwide [2,3]. They have been identified as one of the ‘Evil Quartet’ of major drivers of biodiversity loss worldwide [4] and highlighted in the Millennium (2005) [5] and UK National Ecosystem Assessments (2011) [6]. IAS are the focus of Target 5 of the EU 2020 Biodiversity Strategy, including EU regulation 1143/2014 on management of invasive alien species [7]. The direct costs of invasive alien species have been estimated to be approximately US $1.4 trillion, approximately 5% of global GDP [8], with annual costs of £1.7 billion within Britain alone [9].

Species that arrive in a new country may not establish and only a small fraction of those that establish become invasive [10–13]. Species which become invasive in one area may fail to establish in another, and accurate prediction of the timing, effects and identification of species which may become IAS is not currently possible, despite many attempts [14–23].

This lack in predictive ability to identify which species will become IAS means that regulatory efforts focus on preventing the arrival and establishment of all non-native species. In England and Wales, for example, Section 14 of the Wildlife and Countryside Act makes it illegal to release, or to permit to be released, any animal which is not a resident of, or regular visitor to, Great Britain (http://www.legislation.gov.uk/ukpga/1981/69/section/14).

Hulme *et al*. (2008) [3] identified six distinct pathways by which species may spread beyond their native ranges: 1) deliberate release; 2) unintentional escape; 3) unintentional contaminant of another commodity; 4) unintentional stowaway on transport; 5) natural dispersal aided by human-made corridors; and 6) unaided natural dispersal. In line with the 2011–2020 Convention on Biological Diversity Strategic Plan for Biodiversity, most countries have enhanced their border controls in order to reduce the number of species and individuals arriving via pathways 1–4 [24]. Natural dispersal from a nearby introduced population (pathways 5 & 6) is very difficult to police, therefore these pathways are likely to become proportionally more important in the future as the number of arrivals from pathways 1–4 decreases.

Good dispersal ability has often been found to be an important trait associated with successful establishment and invasion, particularly in insects [25–27], which enables rapid spread beyond the original point of introduction. This is likely to be particularly important for species invading new areas by means of natural dispersal from an introduced population (pathways 5 & 6).

Such a species is the Harlequin ladybird *Harmonia axyridis* (Pallas). Native to eastern Asia, it has been widely introduced outside its native range as a biological control agent and has since spread rapidly to colonise North America, much of Europe, several South American countries, and parts of both northern and southern Africa [28]. The species is a strong flier, actively dispersing over several kilometres to overwintering sites each year [29,30]. It has been observed as high as 1100 m above ground level, moving at 60 km/h and it is able to fly up to 2 h [31]. It was never officially introduced into the UK, although it was repeatedly introduced into several different countries in Continental Europe [25,32,33]. Despite this, the species established itself in Britain in 2004 [34], demonstrating its dispersal ability by moving an estimated 105 km per year northwards and 145 kilometres westwards from 2004 to 2008 [32,33].

Once the species was in Britain, it spread northwards and westwards from the arrival point in the south-east, against the prevailing south-westerly wind direction. For this reason, passive wind-borne transport has been considered unlikely to play a major role in its spread [33,34]. However, as the UK is an island, cut off from the rest of the continent of Europe by the North Sea and English Channel, it has been suggested that the original arrival may have been assisted by wind events [34]. This association between atmospheric events and the arrival of this species has been previously reported [31], but it has not been tested using atmospheric models.

Here, we investigate whether the arrival of an invasive ladybird, *H*. *axyridis*, from continental Europe to Great Britain was assisted by atmospheric events, using a chemistry transport model (CTM) and a numerical weather prediction (NWP) model.

CTMs are mathematical-physical models, which are dedicated to the computation of how particles or gases are transported through, dispersed in, transformed in, and removed from the atmosphere. They are used to forecast air quality [35], support decision-making e.g., in nuclear power plant accidents [36], and in addition, they are useful tools to predict pollen concentrations [37] or migrations of pest insects [38]. Here we are extending their use to analyse the spread of invasive species (small winged insect in this case) through the atmosphere. [35], support decision-making e.g., in nuclear power plant accidents, and in addition, they are useful tools to predict pollen concentrations or migrations of pest insects. Here we are extending their use to analyse the spread of invasive species (small winged insect in this case) through the atmosphere.

Weather forecasts are based on NWP models. They describe atmospheric phenomena in grid cells from Earth’s surface to a height of tens of kilometres using weather observations and mathematical-physical equations and algorithms. NWP models in turn serve the weather information for CTMs, or an CTM system may also include NWP models.

As a comparison, we also examined human-mediated routes of import: the association of *H*. *axyridis* records with seaports and airports.

## Material and methods

### Ladybird records

Biological record data was taken from the citizen science UK Ladybird Survey, collated from records submitted by members of the public to iRecord (www.brc.ac.uk/iRecord), the Harlequin Ladybird survey website (www.harlequin-survey.org) and other sightings submitted to the scheme (principally via email). This recording scheme has been active since 1971, with an online survey launched in early 2005 [39]. All records submitted to this recording scheme are verified by a recognised expert via inspection of a specimen or adequate photograph. This dataset is freely available on the National Biodiversity Network at https://registry.nbnatlas.org/public/show/dr695.

*Harmonia axyridis* was introduced as a biological control agent in France from 1982, the Netherlands from 1996 and in Belgium from 1997 [30]. The species was first found in the wild in Europe in France during 1991, but widespread establishment is not recorded in Europe before the Millennium. Established populations were reported in the Netherlands during 2001, Belgium in 2002, and France during 2003 [30]. After 2003, numbers of *H*. *axyridis* rose fast in all three countries [40]. We selected these countries as source areas, because they were the three countries closest to the south-east of the UK, and thus the vast majority of the early British *H*. *axyridis* records, and all three were known to have populations of *H*. *axyridis* in that time period.

Due to the comparatively small size of the countries compared to the resolution of the model and available data, Belgium and the Netherlands were combined to form one source area. France was treated as a separate source area as atmospheric wind events would have needed to blow in different directions in order to deposit individuals of *H*. *axyridis* in the relevant sighting areas from the two putative sources. European *H*. *axyridis* data was taken from publicly-available data on GBIF (available at https://www.gbif.org/species/4989904).

The first contemporaneous records of *H*. *axyridis* in the UK were made in 2004 and news of the species’ arrival and the subsequent launch of the online survey was heavily publicised. This resulted in a large volume of ladybird sightings (of *H*. *axyridis* and a variety of other species) submitted by the general public [41]. The first larvae to be found in Great Britain (indicating successful reproduction and thus supplementation of immigrant adults with locally-reared individuals) were recorded during 2005 [32]. Consequently, in this paper we examine the years 2004 and 2005 (139 and 2081 *H*. *axyridis* records respectively) as the colonisation period for this species in Britain.

To provide an estimate of the relative proportion of *H*. *axyridis* records to the background level of ladybird records submitted over time and ensure that spikes in *H*. *axyridis* numbers were not just good days for recording ladybirds, we compared the *H*. *axyridis* records to records of six widespread and abundant ladybird species (*Adalia bipunctata* (L.), *Adalia decempunctata* (L.), *Calvia quattuordecimguttata* (L.), *Coccinella septempunctata* (L.), *Halyzia sedecimguttata* (L.) and *Propylea quattuordecimpunctata* (L.)) over the same time period (4479 records in total).

To characterise whether the location of *H*. *axyridis* records in 2004 and 2005 in the UK (n = 139) were clustered or randomly distributed with respect to ports and airports, we used Ripley’s K-function from package “spatstat” [42] in the statistical language R. We created two shapefiles, incorporating 59 airports and 31 major ports (Table PORT0103, https://www.gov.uk/government/statistical-data-sets/port01-uk-ports-and-traffic) in England and Wales. To determine whether the *H*. *axyridis* records were similarly clustered with either airports or ports, we used Monte Carlo simulation with random labelling of points and cross K-function [43].

### SILAM

We used the chemistry transport model SILAM (System for Integrated modeLling of Atmospheric coMposition, http://silam.fmi.fi) to simulate the atmospheric movements of *H*. *axyridis*. It has been used to predict the migration of pest insects [38] and thus has a known ability to simulate the atmospheric transport of biological organisms.

SILAM is a meso- to global-scale, mathematical-physical atmospheric composition model which can use both Lagrangian (random walk particle model) [44] and Eulerian (atmospheric transport computed in a grid) [45] approaches. It was originally developed as an emergency transport model of radioactive releases. It is still used for this purpose in both forward and inverse (footprint) mode [46], as well as for several other particulate-modelling purposes, e.g., air quality [47], transport of volcanic ash [48], and numerical pollen predictions [49–51]. It is currently used as the official air quality forecasting tool in Finland [35] and is used operationally amongst other CTMs e.g., in the Copernicus Atmosphere Monitoring Service (CAMS) http://macc-raq-op.meteo.fr/, WMO Sand and Dust Storm Warning Advisory and Assessment System (SDS-WAS, North Africa and Europe) https://sds-was.aemet.es and Air quality forecast for China (MarcoPolo-Panda) http://www.marcopolo-panda.eu/forecast/. SILAM gives similar results to other CTMs e.g., [52–55].

The forward atmospheric transport models investigate where material will be transported to when the source area is known, whereas the inverse atmospheric transport models investigate where the source area is for observed material. Theoretically, forward atmospheric dispersion equations can be used in inverse calculations, only direction of time is negative [44,56]. The forward CTM outputs particle counts, concentration etc. if the source is known well enough or a dispersion area, which describes the area which is affected by the source. Here it is called potential landing area for *H*. *axyridis*. The inverse CTM outputs probability area (~ footprints), not exact numbers, providing the area where the source could be located within. The source or sources can be located at any point within the probability area and users should evaluate if it is possible (e.g., the source of ladybirds cannot be on the sea). Different data-assimilation methods could reduce the probability area, but they require considerable amounts of observations.

**Fig 1:**
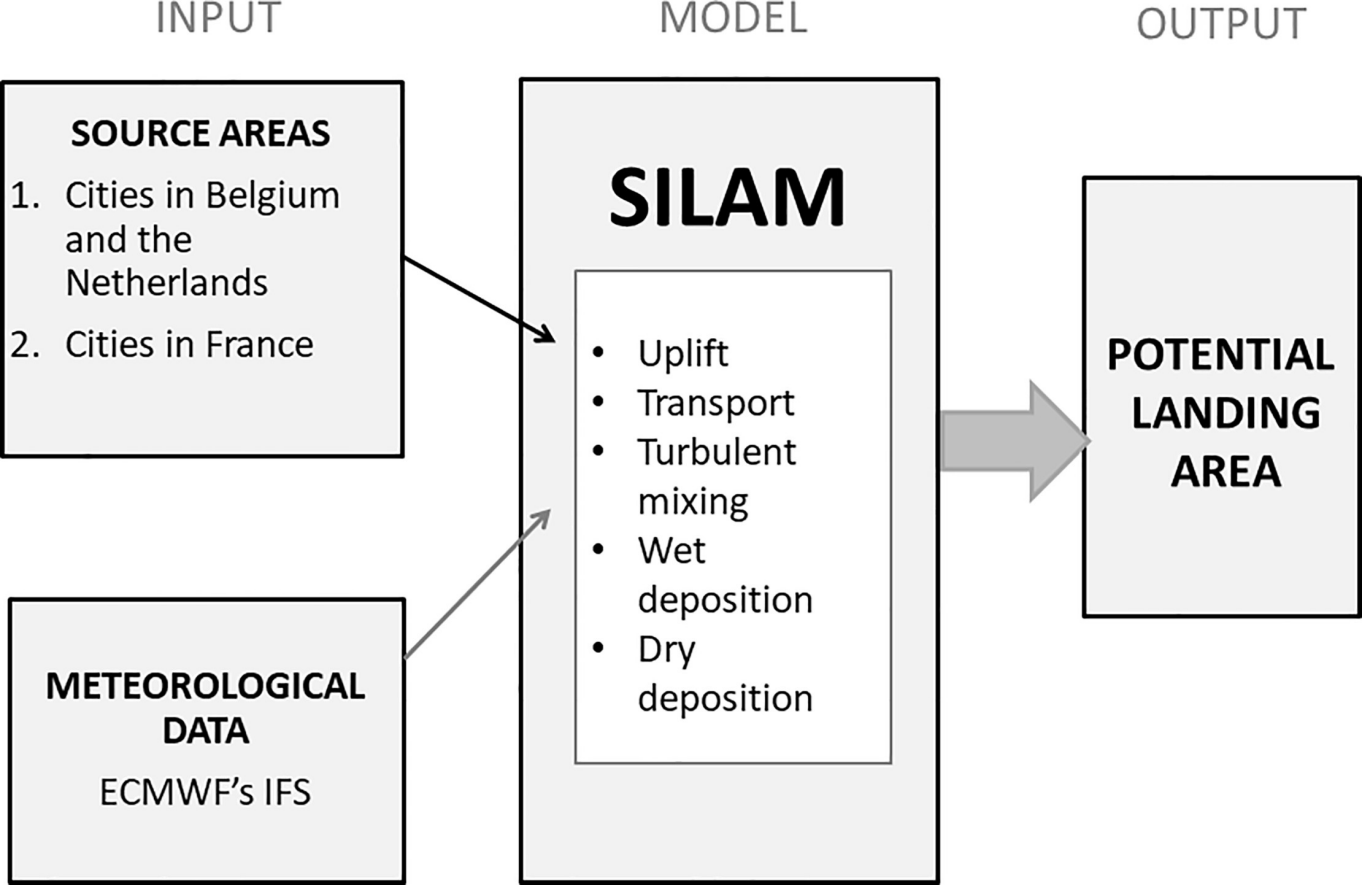
SILAM forward *H*. *axyridis* simulation. As input information the SILAM ATM forward *H*. *axyridis* simulations need source areas and meteorological data from a NWP model. SILAM computes uplift of the insects, transport, turbulent mixing and removal from the atmosphere. The model gives potential landing area of *H*. *axyridis* as a result.

Once the material is emitted to the atmosphere, wind transports particles and gases in the atmosphere, turbulent eddies mix them and rain (wet deposition) and gravitational sedimentation (dry deposition) clean them out. SILAM takes all these processes into account [45] (Fig 1).

Most of the particles (pollen, dust, flying animals, etc.) which are released inside the atmospheric boundary layer stay there while they are in the air, but some of them are able to escape and go higher. The height of the boundary layer depends on weather, but in mid-latitudes it is typically the lowest 1000–2000 metres of the atmosphere.

Insects generally fly inside the atmospheric boundary layer. This means that CTMs, dedicated to the modelling of particles originating in this layer, are also useful tools to simulate long-distance movements of small insects. As relatively weak fliers, most small insects largely follow the prevailing wind direction and are capable of only slightly affecting their flight directions [57]. Migratory species of moth have been shown to be able to exploit different wind speeds and directions at different heights to considerably extend their dispersive range [58,59]: although *H*. *axyridis* is not known to have this ability, the particle travel distance should be taken as a conservative estimate of the potential maximum distance travelled for the species. The insect’s own velocity can cause small inaccuracies to the model results compared purely passive transport of particulate matter (PM), but even insects engaged in migration have their direction of movement largely determined by the wind direction [60–65].

*Harmonia axyridis* is not known to be a migratory species, or to be able to undertake long-distance directed flights, so we modelled the dispersal of *H*. *axyridis* individuals as inert coarse-particulate particles travelling with the air masses. Both *H*. *axyridis* and the coarse PM10 particles (particulate matter with a diameter smaller than 10 μm) stay about same time (some hours) in the atmosphere. Insects modelled as PM still take into account turbulent eddies, rain and limited flight time, while simple trajectory analysis would give only rough estimate of where the insects come from or where they are going.

The SILAM modelling process requires a source area for the modelled (Fig 1). *Harmonia axyridis* is known to reach a high abundance in urban areas [66], so we took as source areas the larger cities near the north coasts of Belgium (Antwerp, Gent, Bruges, Brussels), the Netherlands (Amsterdam, Hague, Rotterdam) and France (Dunkirk, Lille, Calais, Amiens, Le Havre, Dieppe).

The ECMWF’s operational numerical weather prediction (NWP) model data (Integrated Forecast System–IFS: https://www.ecmwf.int/) served as a source of weather information for SILAM (Fig 1) both in forward and footprint computations. The NWP model is global and thus it covers the whole dispersion area we were interested in (10.5°W-10°E, 45°N-60°N). We used the data as 3-hour time steps (+3 h, +6 h, +9 h and +12 h forecast lengths) and with a square grid size of 0.225° and 21 NWP-vertical levels from ground to over 5 km. The most important weather parameters used were 3D-winds (the transport of ladybirds) and rain (influences deposition of ladybirds from the atmosphere).

The SILAM *H*. *axyridis*-simulations shown here were computed using Eulerian-SILAM using the same grid as in the NWP model (0.225° x 0.225°, i.e. about 15 x 25 km). We used a time step of 15 minutes in the SILAM atmospheric transport calculations, which contains uplift of *H*. *axyridis* individuals, transport with winds, turbulent mixing and wet and dry depositions (Fig 1). SILAM gives potential landing area as a result (Fig 1).

We carried out modelling from the 1^st^ June 2004 (the first month with records of *H*. *axyridis*) until 1^st^ October 2004 (the ladybirds largely cease outdoor activity and enter overwintering sites around this time). For 2005, we modelled 1^st^ April-1^st^ October in order to capture both the spring and autumn dispersal periods, as well as the summer activity period. SILAM source points in Europe were modelled as continuously releasing ladybird particles every day across the two years examined, between 5 am and 6 pm, UK time.

The ladybird’s habit of overwintering inside buildings causes a spike in records in late autumn as they are noticed by householders. There was no way to reliably split these records from those of ladybirds outside, which might be affected by atmospheric events, so we excluded records from the overwintering period (1^st^ Oct-31^st^ March). We did not model past 2005 as the establishment and rapid spread of the species would have made the distinction between newly-arrived immigrant individuals and existing residents impossible to quantify.

Inverse SILAM (‘footprints’) were computed 72 h backwards from the ladybird observations in 2004. Source points (detection points) and direction of calculation (backward in time) were different than in the forward SILAM simulations, but otherwise the model setup was same. We expected that ladybirds arrived at the earliest one day before observations, latest in the middle of the observation day (e.g., obs. 15/8/2004, “collection time” 14/8/2004 00 UTC-15/8/2004 12 UTC), so the collection time was 1.5 days and simulated period (dispersal time plus collection time) was 1.5–3 days backward. However, the SILAM-footprints showed in many cases that the ladybirds arrived earlier than 1.5 days before observations. Thus, we also computed 10 days inverse simulation for the record on the 30^th^ June, 2004, which is the earliest UK record in 2004. There the collection time was taken as 10 days as well.

The model output information was produced in 10 km square grid cells. The area extended from the 45°N to the 60°N and from 10.5°W to 10°E to cover the UK, Ireland, Belgium, the Netherlands and part of France, Germany, Denmark and Norway. In case of the forward simulations, the output was given as a daily average, whereas in case of the footprints it was hourly.

### Associating SILAM events with *H*. *axyridis* arrivals

We used Generalised Linear Models (GLMs) to determine whether SILAM-predicted atmospheric events from source populations were associated with records of *H*. *axyridis*. If these ladybirds were arriving from cross-channel atmospheric events, we would expect to see the number of records of *H*. *axyridis* to be better associated with SILAM-predicted atmospheric events when close to the south-east coastline, with the association reducing with greater distance from the coast. Previous work [67] has found *H*. *axyridis* able to fly at up to 60 km/hr at high altitudes, and to fly for at least two hours. To allow for any extra flight time (Jeffries *et al*. [67] stopped monitoring at a two-hour flight time cut-off), plus any short-range flights during the collection period, we split the ladybird dataset into two, and compared *H*. *axyridis* record numbers within 200 km of the continental coastline to numbers collected further than 200 km from the continental coastline. For both datasets we determined the daily frequency of *H*. *axyridis* and the daily frequency of the 6 most commonly recorded ladybirds. A bound vector of daily frequency of *H*. *axyridis* records and the daily frequency of common ladybirds was used as the response variable in GLMs with binomial error structures. We calculated the maximum value of SILAM per day from both source populations for a 7-day window for 51°N 1°E (West Kent coastline) around the ladybird record days and used this as an explanatory variable in the models. The maximum value was used, because the SILAM *H*. *axyridis* footprint studies showed that it is not likely that the recorded date is the date of arrival, but an earlier day within about one week. We were also interested in the association of records with month, and how this differed between years, therefore a combined value of month and year (i.e. 2004.6 to represent June 2004) was also included as an explanatory value.

## Results

### Spatial autocorrelation of airports, ports and *H*. *axyridis*

**Fig 2:**
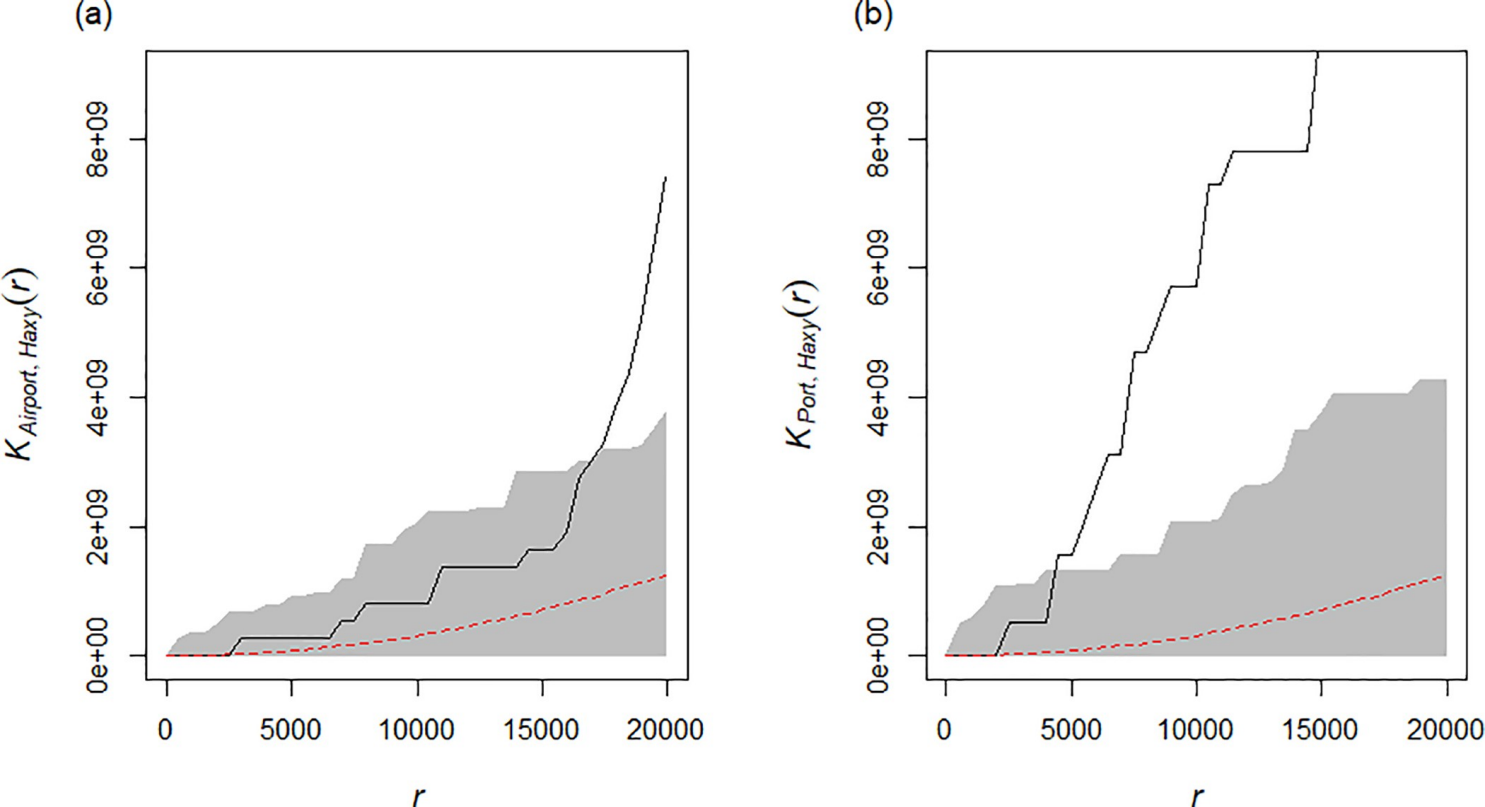
Monte Carlo K-cross simulations for *H*. *axyridis* records. Monte Carlo K-cross simulations (n = 1,000) for *H*. *axyridis* records in 2004 and 2005 and airports (a) and ports (b). The red dotted line represents what would be expected with the points were randomly distributed; the grey area around this represents the confidence envelope from the Monte Carlo simulations. The black line represents the observed K values; where this line falls within the grey area the points can be described as not associated; however, outside the grey area, the points can be considered to be associated. On the x-axis, r represents cluster distance in metres.

Bivariate Ripley-K functions suggested that the location of *H*. *axyridis* records in the first two years of arrival were not associated with airport locations in England and Wales at cluster distances of < 17 km (Fig 2A). *Harmonia axyridis* records were also not associated with port locations in England and Wales from 0–5 km cluster distances, but showed some association at greater distances (Fig 2B).

### SILAM events

**Fig 3:**
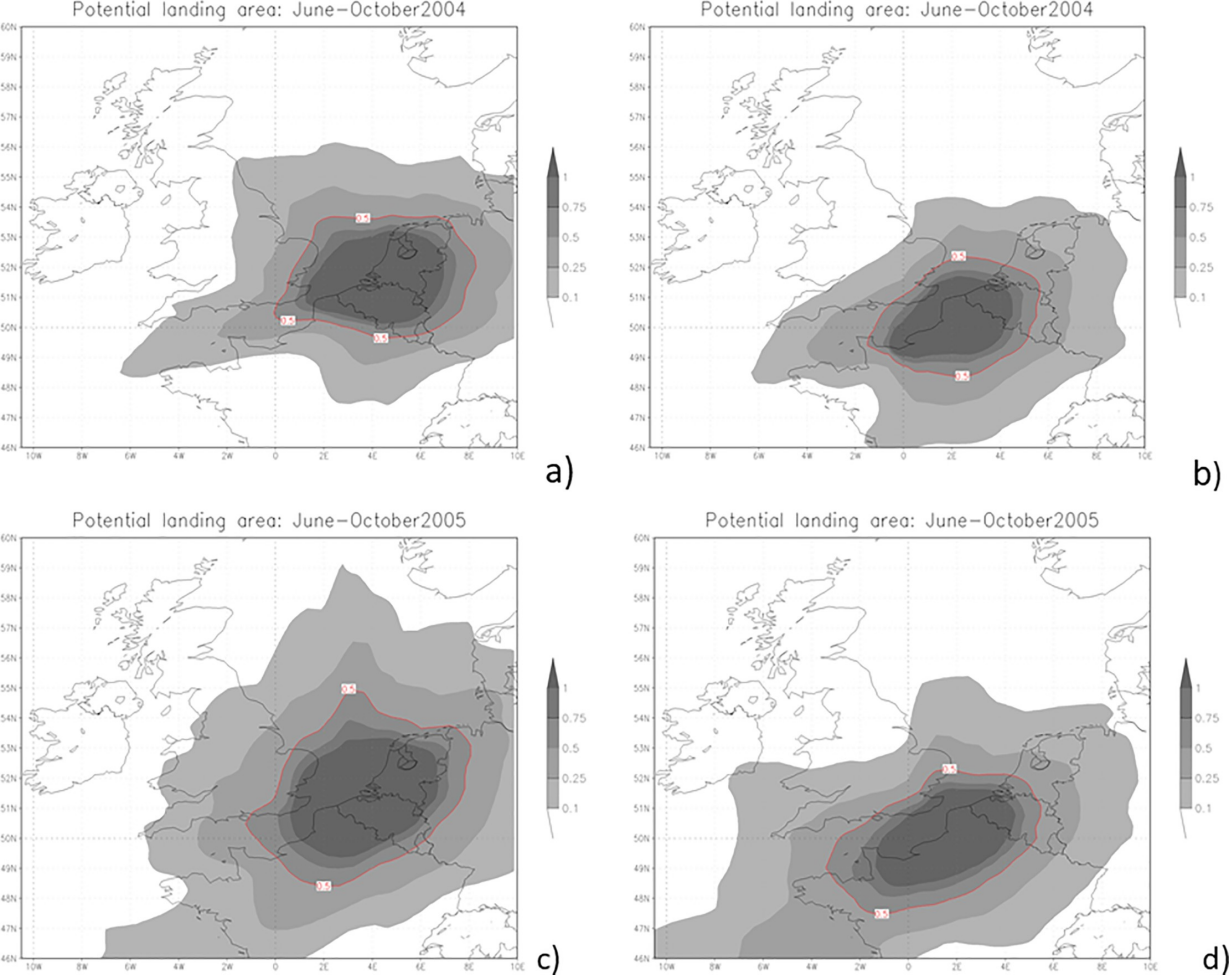
SILAM potential landing area predictions. SILAM potential landing area predictions for June to October 2004 (a,b) and 2005 (c,d) for Netherlands & Belgium combined (a,c) and France (b,d). Higher values (inside contours) suggest a high probability of arrival in the UK from source populations due to atmospheric events. Background maps OpenStreetMap contributors.

Air currents from source populations in France, Belgium and the Netherlands could feasibly have transported *H*. *axyridis* individuals across the English Channel, as the first records are located in the south-east, in line with the SILAM predictions (Fig 3). These predictions suggest that 2005 may have been more favourable for the migration of ladybirds than 2004, as the events were stronger and more frequent in the latter year, especially from Belgium and the Netherlands, where the population appears to be larger. More favourable winds in the later year can be seen also in Fig 3, where the average potential landing area is larger in 2005 than in 2004.

A more detailed examination showed that air flows are suitable for ladybirds to come from Belgium and the Netherlands, on average, once a week (13.9% of days) during June-September 2004, but from France 1–2 times a week (20.5% of days). In 2004, the best month to fly over the English Channel was September (23.3% of the days from Belgium and the Netherlands and 20% from France were suitable). In April-September 2005 a higher proportion of days were suitable for ladybirds to cross the English Channel: 17.6% of the days from Belgium and Netherlands and 22.0% of the days from France. September 2005 was particularly favourable for migration from the continental Europe to the UK, with conditions suitable for migration from Belgium and the Netherlands almost every four days (23.3%) and more than every three days (33.7%) from France. This coincides with observations of *H*. *axyridis* becoming more common.

We computed the inverse SILAM (footprints) for all seven UK records of *H*. *axyridis* observed before October 2004. The SILAM footprints suggested that France could be the source area for three of the seven records, Belgium or Netherlands for two of the seven, and the source area is unclear for the remaining two records.

**Fig 4:**
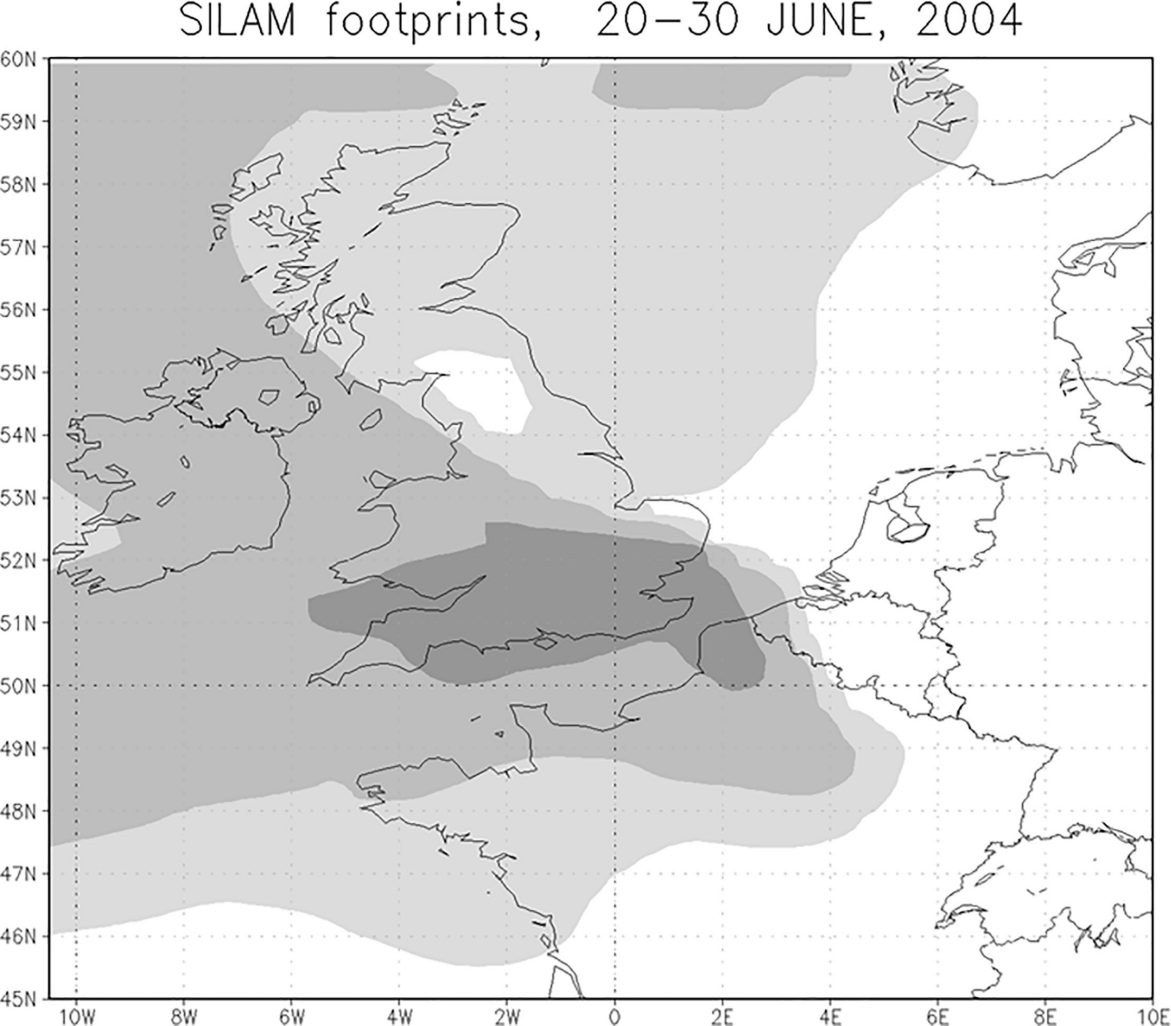
Inverse SILAM simulation. Inverse (footprint) SILAM simulation 10 days backwards from *H*. *axyridis* record in Kent (51.3° N, 0.9°E) on 30^th^ June, 2004 expecting continuous collection time. The model demonstrates the potential source areas for the record, with darker areas indicating a greater probability of the source location, though it should be noted that ladybirds could only have originated from terrestrial areas. Background map OpenStreetMap contributors.

It is likely that the recorded individual had been present for some time before it was found, therefore 1.5 days of collection time only provides a limited snapshot. Therefore we carried out a more detailed analysis of the potential arrival mechanism for the first 2004 UK record of *H*. *axyridis*, on the 30^th^ June 2004 from Faversham, Kent. Fig 4 shows an example of mapping the probable area of origin (cumulative, backward probability area) of particles arriving at Faversham (51.3° N, 0.9°E) for the ten days immediately preceding 30^th^ June, 2004. This suggests that the *H*. *axyridis* observed on 30^th^ June most likely came to the UK during morning hours on 26^th^ June, from France. Several days before and after that short moment of opportunity winds did not blow in a favourable direction from continental Europe.

**Fig 5:**
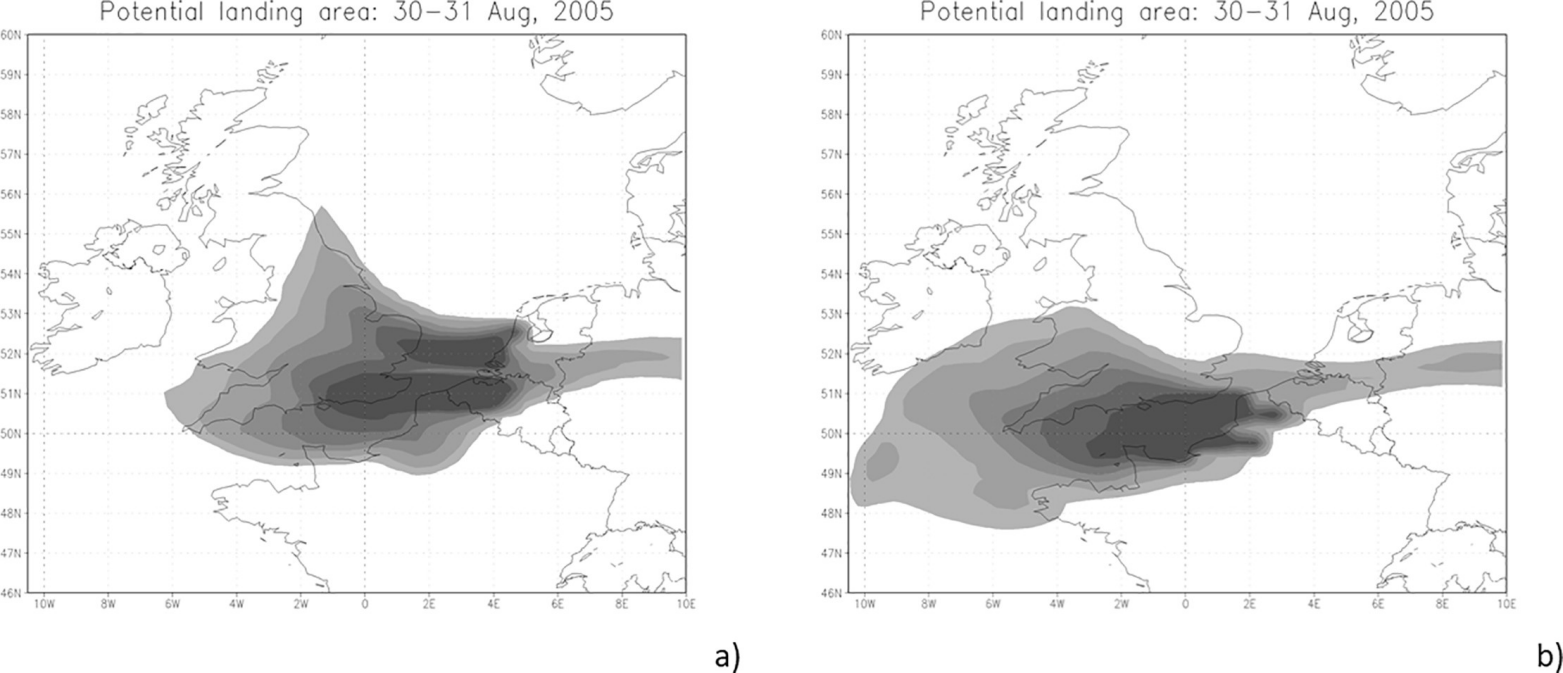
SILAM forward simulations. SILAM forward simulation on 30^th^ August, 2005, 6 UTC - 31^st^ August, 2005, 6 UTC. Source areas locate in a) Belgium and the Netherland and b) in France. Background maps OpenStreetMap contributors.

Another example is for 2005. On 1^st^ Sept, 2005 there were 13 *H*. *axyridis* records, 72% of all observed ladybirds in the UK that day, all of which were within 200 km of the coastline. The SILAM forward simulations from Belgium and the Netherlands (Fig 5A) and from France (Fig 5B) shows that in this case the source was more likely to be located in Belgium and the Netherlands than in France. The case was also clearly stronger compare to case in the end of June, 2004. Thus, the weather provided an efficient path for *H*. *axyridis* to arrive in the UK.

### Association of *H*. *axyridis* records and SILAM events

**Fig 6:**
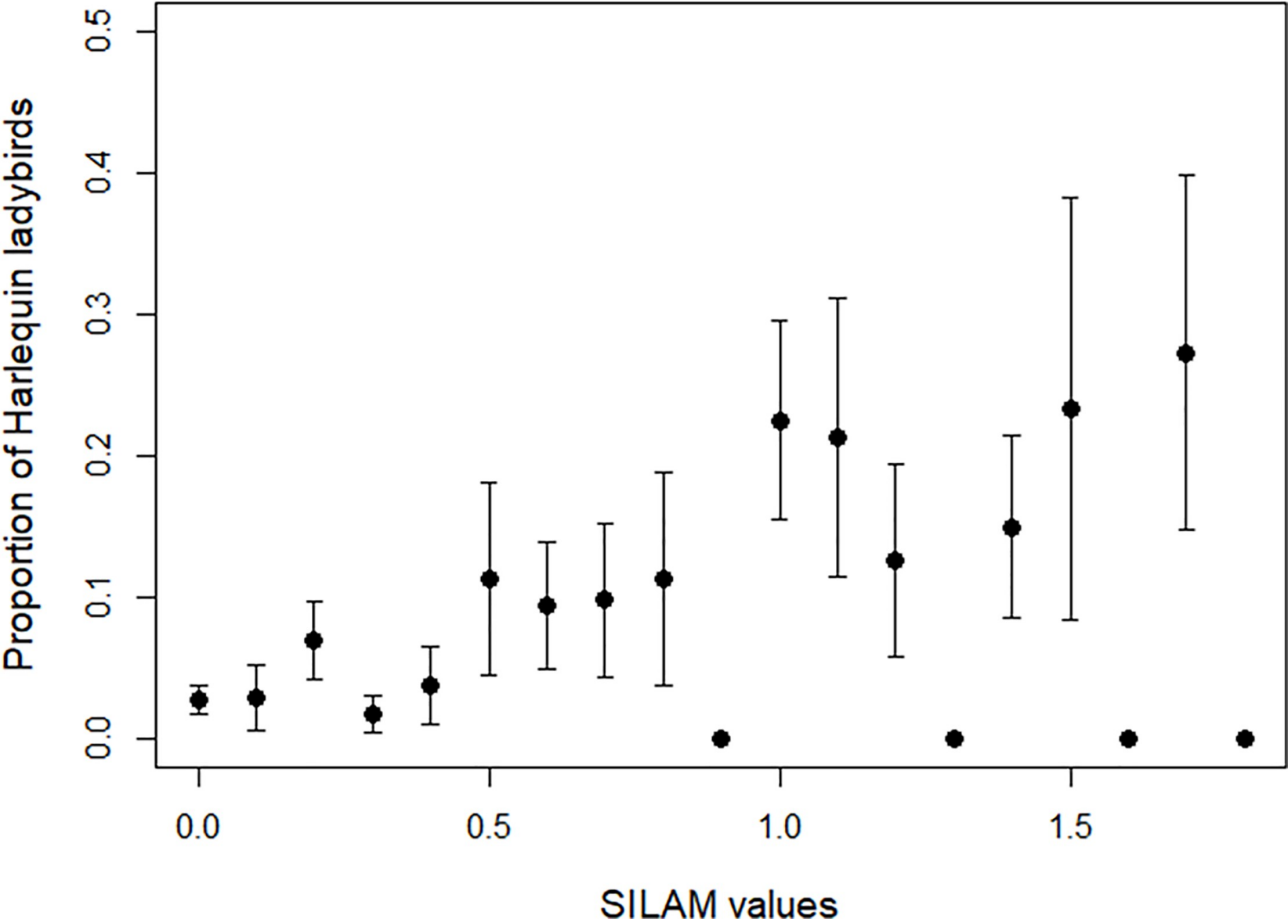
Mean proportion of *H*. *axyridis* records to common species within 200 km from continental coastline with SILAM atmospheric event values. SILAM events have been rounded to nearest 1 decimal place. Error bars represent ±1 S.E.

At distances less than 200 km from the continental coastline, we found that there was an association between the daily maximum SILAM values and the proportion of *H*. *axyridis* records (Quasi-binomial GLM: correlation coefficient: 0.42; LR: 4.22, p = 0.04). With greater values of SILAM, higher proportions of *H*. *axyridis* records were submitted compared to common native species (Fig 6). There was significant variation in the proportion of *H*. *axyridis* records to the combined total of the six common native species over time (Fig 6; LR: 31.94, p < 0.001), with a mean proportion of 0.10 *H*. *axyridis* to native species from June to September 2004, but increasing to a mean proportion of 2.53 *H*. *axyridis* to native species from April to September 2005.

In contrast, at distances greater than 200 km from the continental coastline, we found that there was no association between the daily maximum SILAM values and the proportion of *H*. *axyridis* records (Binomial GLM: correlation coefficient: 0.64; LR: 2.79, p = 0.09). However, there was significant variation in the timings of these records (Month & Year: LR: 38.75, p < 0.001), with higher mean proportions of *H*. *axyridis* to common native species recorded in May 2005 (0.42), June 2005 (0.43) and September 2005 (0.43) than in July 2005 (0.29) and August 2005 (0.26).

## Discussion

The spread of *H*. *axyridis* is known to have been assisted by anthropogenic introductions, and some have also alluded to possible atmospheric-assisted dispersal [33,34]. Here, we demonstrate that atmospheric events are a viable, likely and detectable means for this species to have dispersed over a large natural barrier between continental Europe and the UK in 2004 and 2005.

According to SILAM simulation, *H*. *axyridis* could fly from France to the UK (Kent and Essex) within 1–3 hours, which is a feasible flying time for ladybirds ([68] p.348, [31]). It would take longer (4–6 hours) to reach more northern locations like Suffolk and Norfolk from France.

We found some clustering of records of *H*. *axyridis* with port and airport locations at a regional scale, with all records and a large number of these transport hubs in the south-east of England, near the European continent. We found no clustering at small scales, with no evidence of any increase in numbers of *H*. *axyridis* above the average in the immediate vicinity of airports (up to 17 km radius) or ports (up to 5 km). There was no evidence of *H*. *axyridis* sightings at or near ports or airports away from the southeast of England, despite the existence of multiple transport pathways from areas inhabited by the species in continental Europe to UK ports & airports outside south-eastern England. It is impossible to rule out the role of anthropogenic transport entirely: indeed, we would not wish to do so: there is considerable anecdotal evidence of ladybirds being moved on ships and other motorised transport [39]. It is also possible for individuals of a species to be transported away from their immediate arrival point in a new country (i.e. the port/airport) before being released to the environment, for example sealed in a parcel until unpacked, and this is known to be one of the invasion routes for *H*. *axyridis* [69]. However, it seems unlikely that purely anthropogenic transport would produce the observed arrival pattern, with no clustering around transport hubs within the atmospheric model’s potential landing area, and no sightings away from this potential landing area despite the presence of multiple major ports & airports receiving ships/aircraft from areas with populations of *H*. *axyridis* (in many cases the same areas providing the potential source populations for transport hubs in the south-east of England). This, combined with the correlation in timing between atmospheric events and ladybird records, suggests that atmospheric transport is a likely primary method for the species’ arrival.

*Harmonia axyridis* has been reported in 53 countries outside its native range. When examining the spread of the species, it is striking that island nations, particularly those that have strict biosecurity systems to detect and detain non-native species at the border (e.g. Australia, Cyprus, Iceland, and Malta) have been largely unaffected by *H*. *axyridis* [28,70]. As controls become stricter on anthropogenic transport pathways, the number of individuals imported this way is likely to decrease. These controls will not affect natural cross-border dispersal of individuals from an invasive population (i.e. Hulme’s [3] pathways 5 & 6, organisms moving without anthropogenic assistance). Consequently natural cross-border dispersal, including the wind-assisted long-distance dispersal examined here, is likely to become proportionally more important as a means of spread for non-native invasive species. Our analysis indicates that it is possible for *H*. *axyridis* to be carried across the English Channel, from where it is known to have successfully established. Other small winged animals are likely to be able to undertake similar wind-assisted dispersal to the UK and other island nations. Indeed, over the last two decades, there have been many new species that have established in the UK: some which are flightless and thus must have arrived through anthropogenic transport, such as flatworms [71], but also several winged species, including some good fliers, such as the 20 new species of moth [72], but also a group of several others which, although capable of flight, are more often associated with short-range dispersal rather than long-distance migration. This includes the ladybirds *Henosepilachna argus*, *Rhyzobius chrysomeloides*, *Rhyzobius lopanthae*, *Rhyzobius forestieri*, and *Scymnus interruptus* [73].

Wind-assisted passage from continental Europe may be a particularly important route for species that are more adapted to passively utilising wind currents to disperse, such as juvenile spiders. Many spider species disperse as juveniles by ballooning, where immature individuals take off by spinning long threads of silk, allowing them to be blown around on the air currents. The ballooning behaviour of Wasp spiders *Argiope bruennichi* [74], combined with favourable atmospheric events (no rain, the wind blowing from the right direction, and the presence of vertical upwards air movements) during the species’ dispersal period may well have been instrumental in the arrival of this species from continental Europe to the UK in the 1990s. We suggest that the exhibition of traits associated with greater dispersal potential via atmospheric events, such as ballooning behaviours or the production of macropterous forms (e.g., for the orthopteran *Metrioptera roeselii* [75,76], together with frequent atmospheric events facilitating long-distance dispersal, is likely to be particularly important for saltatorial population expansion across waterbodies or other large-scale barriers to spread. Once introduced, other factors, such as climate, may play a more influential role in species’ spread each year [77,78].

Our results demonstrate that *H*. *axyridis* colonising the UK via atmospheric events had more opportunities to have originated from France, as southerly winds are more common than easterlies. However, from the available evidence [79], populations of *H*. *axyridis* appear to be larger in Belgium than in France [79] and so, despite fewer atmospheric events originating from Belgium, each event has a higher likelihood of bearing ladybirds. This probable influx of individuals from multiple sources has likely contributed to the later successful establishment and spread via intraspecific but interpopulational admixture [80,81].

In many invasion events (but not all), source populations can be identified using genetic methods [82]. However, this approach may be affected by sampling errors [83], and it is not predictive in terms of arrival dates or methods. For species known to disperse aerially, atmospheric modelling may provide a rapid assessment of areas with a high likelihood of arrival of the species, potentially in a real-time fashion (as is currently carried out for weather and pollen forecasts, for example). Where speed of detection is required, for example to eliminate potential invasions of injurious species, this predictive approach would allow the warning and priming of survey networks, for instance by circulating photographs of the potential arrival to citizen scientists and biological recorders such as lepidopterists running light traps, with a request for any records which might arise.

One major limitation of this approach is that biological records used within the model, and also those used in model evaluation, need to be relatively comprehensive. Biological recording schemes have increased in presence and reach in the last decade, particularly with the use of online tools, but for novel species there may be a lag between arrival and sufficient records to build an accurate picture of the introduction event. In the case of *H*. *axyridis*, although the species was first identified and reported to the Ladybird Survey in October 2004, when the sighting was publicised several earlier records were submitted, including three specimens from 2003 which had been missed at the time or which had remained unidentified [34]. As a volunteer survey with (in 2004) relatively limited participation, the network was not particularly sensitive to detecting low numbers of a new species. However, it should be noted that *H*. *axyridis* is a large and obvious species which often lives in close proximity to people and is apparent even to non-entomologists.

While many countries have tightened their airport security in response to increased knowledge of IAS [24], finding individuals of small species is still challenging, particularly if in personal luggage or live plants. Moreover, individuals assisted by unpredictable atmospheric events to cross large natural barriers bypass these security measures; therefore, a more integrated approach to IAS management should include tracking storm events and subsequent records. This should include the development of predictive models of periods of high risk of arrival of airborne species, and increased, targeted surveillance (including working with volunteer recorders) carried out. *Harmonia axyridis* has had a dramatic impact on native ladybirds in the UK, eating the larvae of many species [28,32,84]; if this invasion had been detected and managed appropriately in the early stages, this may not have occurred.

We hope that the ongoing growth in biological recording, with increasing availability of resources and speed of communication of sightings, will make recording schemes a better real-time early warning system for novel arrivals. More recorders, more and faster access to verifiers, better platforms for timely mass publication of sightings (such as social media), along with greater and more accurate public awareness of novel, potentially harmful species, such as the Asian hornet *Vespa velutina* or Asian Longhorn beetle *Anoplophora glabripennis*, all make speedy detection, identification and dissemination of new species both more possible and more likely. The GB Non-native Secretariat has a list of many potential invaders [83]; if these species are targeted for public awareness campaigns, they may be detected before they establish. One such project currently holding off a full-scale invasion is that concerned with the Asian hornet. In 2004, the Asian hornet *Vespa velutina* was accidentally introduced to south-west France and has spread rapidly, with sightings in Spain [85], Portugal [86], Belgium [87] and Italy [88]. A predator of European honeybees *Apis mellifera*, arrival of this species has been associated with economic impacts on apiculture and pollinator decline [89]. It was first recorded in the UK in 2016 [90,91] and has been found across the south of England from Cornwall to Kent [91]; using storm events to predict areas in need of enhanced nest surveillance may help to reduce the likelihood of this species becoming established in the UK.

Atmosphere is a viable route for invasion, over which we have no control. Given current uncertainty about future climate change, greater frequency of storm events for example, could increase or decrease risk of invasion via this pathway.
